# The impact of sugammadex versus neostigmine reversal on return to intended oncological therapy-related outcomes after breast cancer surgery: a retrospective cohort study

**DOI:** 10.1186/s13741-025-00591-z

**Published:** 2025-09-02

**Authors:** Nicolas Cortes-Mejía, Juan Jose Guerra-Londono, Lei Feng, Jose Miguel Gloria-Escobar, Heather A. Lillemoe, Gavin Ovsak, Juan P. Cata

**Affiliations:** 1 Department of Pain Medicine, The University of Texas MD Anderson Cancer Center, Houston, TX USA; 2 Department of Anesthesiology, Henry Ford Health System, Pain Management, & Perioperative Medicine, Detroit, MI USA; 3 Department of Anesthesiology and Perioperative Medicine, The University of Texas MD Anderson Cancer Center, Houston, TX USA; 4 Department of Biostatistics, The University of Texas MD Anderson Cancer Center, Houston, TX USA; 5 Departmento de Medicina de Enlace, Clínica Astorga, Medellín, Antioquia Colombia; 6 Department of Breast Surgical Oncology, The University of Texas MD Anderson Cancer Center, Houston, TX USA; 7 Anesthesiology and Surgical Oncology Research Group, Houston, TX USA

**Keywords:** Breast cancer, Mastectomy, Return-to-intended oncological therapy, RIOT, Sugammadex

## Abstract

**Background:**

Early return to intended oncological therapy (RIOT) after cancer resection is a determinant for long-term oncological outcomes. Sugammadex is increasingly used to reverse the muscle relaxant effect of rocuronium during general anesthesia. It has been shown to improve early postoperative outcomes, but its impact on RIOT is unknown. This study tested the hypothesis that the administration of sugammadex during mastectomy for nonmetastatic breast cancer resection would be associated with better RIOT-related outcomes compared with neostigmine.

**Methods:**

Women ≥ 18 years who required mastectomy for nonmetastatic breast cancer resection from 2015 to 2022 were included in the retrospective study. They were grouped according to the administration of sugammadex or neostigmine. The study outcomes included time to RIOT, the incidence of RIOT at 90 and 180 days, length of hospital stay, and rate of 30-day hospital readmission. A multivariate analysis was conducted to test the association between sugammadex use and RIOT-related outcomes.

**Results:**

Of 888 patients who met the study criteria, 319 received neostigmine and 569 received sugammadex. Sugammadex patients achieved RIOT at 90 days in 81.9% of the cases, whereas 70.8% of neostigmine patients were able to achieve RIOT (*P* < 0.001). Similar results were found for RIOT at 180 days (85.8% vs. 76.8%, respectively; *P* < 0.001). Sugammadex patients achieved RIOT faster than neostigmine patients (37 days, 95% *CI*: 35–41 days; *P* < 0.001). However, the multivariate analysis for RIOT initiation and time to RIOT did not show statistically significant differences.

**Conclusion:**

The administration of sugammadex, compared with neostigmine, is not associated with significant improvements in RIOT-related variables after breast cancer surgery.

## Introduction

Breast cancer is the most frequently diagnosed malignancy in women, accounting for up to 32% of all cancers diagnosed in the USA (Siegel et al. 2024). In 2024, 310,720 new cases of breast cancer are expected to be diagnosed in the USA, along with approximately 42,250 deaths due to this malignancy (Siegel et al. 2024).

Despite recent improvements in neoadjuvant strategies and surgical techniques, up to 4.7% of breast cancers recur locally, 3.0% regionally, and 15% as metastatic disease, with variable rates depending on tumoral biology and the treatment received (Wang and Wu 2023; Maaren et al. 2019). Considering this, the relevance of adjuvant treatments, including endocrine therapy, chemotherapy, and radiotherapy, has been highlighted in recent major randomized control trials in different populations of breast cancer patients (Kunkler et al. 2023; Slamon et al. 2024; Whelan et al.., 2023). Furthermore, the rate of adjuvant therapies use in women with breast cancer can exceed 50% (Rogowski et al. 2023; Zhai et al. 2020). This is evidence that thousands of women are candidates for adjuvant therapies.

Return to intended oncological therapy (RIOT) has emerged as a novel metric for patients requiring adjuvant therapies after surgery for primary or metastatic cancer (Hayden et al. 2020; Kim et al. 2016; Lillemoe et al. 2019; Finnerty and Buggy 2020; Cortes-Mejia et al. 2024). Evidence shows that patients with breast cancer who are unable to receive adjuvant therapies or experience a delay in their initiation have worse oncological outcomes (Chavez-MacGregor et al. 2016; Biagi et al. 2011; Cold et al. 2005; Ke-Da et al. 2013). Although perioperative anesthetic interventions, including regional anesthesia or the general anesthesia technique, have not been shown to improve oncological outcomes, a recent randomized controlled trial demonstrated a beneficial impact in RIOT (Hayden et al. 2020; Sessler et al. 2019; Xu et al. 2021; Du et al. 2021; Falk et al. 2021). Hayden et al. showed that women with gynecological cancers who were treated with intraperitoneal ropivacaine instillation for 72 h had a shorter time to RIOT (20 ± 29 days) than those who received placebo (21 ± 40 days) (Hayden et al. 2020). Other intraoperative interventions such as the administration of muscle relaxants have been associated with an impact on RIOT. Niu et al. showed a negative association between high dosages of atracurium and cisatracurium and time to RIOT in patients with gastric cancer undergoing surgery with curative intent (Niu et al. 2020).

In 2015, the Food and Drug Administration approved sugammadex as the first cyclodextrin agent to specifically reverse the effect of rocuronium and vecuronium (Kovac 2009). Since its approval, there has been an increased use of the drug in the USA and in our institution (Dubovoy et al. 2020). Studies have shown that sugammadex, compared with neostigmine, causes a shorter recovery from neuromuscular blockade, a reduction of up to 30% in the composite rate of postoperative pulmonary complications, and faster discharge rates without accelerating bowel function (Ruetzler et al. 2022; Suleiman et al. 2023; Piccioni et al. 2024; Olesnicky et al. 2024; Azimaraghi et al. 2023; Postaci et al. 2024).

Currently, there is a gap in knowledge regarding the impact of sugammadex, compared with neostigmine, on RIOT. To address this gap in knowledge, we conducted a retrospective study on women who underwent mastectomy at a large tertiary cancer center. Our objective was to determine if there were differences in RIOT rates, time to RIOT, and postoperative complications in women who underwent mastectomies and received neostigmine or sugammadex intraoperatively. We hypothesized that sugammadex, compared with neostigmine, could increase RIOT rates and decrease the time to RIOT after mastectomy for breast cancer by accelerating recovery times and reducing postoperative complications (Niu et al. 2020).

## Methods

The University of Texas MD Anderson Cancer Center Institutional Review Board reviewed and approved this research protocol (IRB no. 2023–0361) on June 28, 2024. Given the study’s retrospective nature, a written consent waiver was granted. The authors conducted this study according to the ethics principles covered by the Helsinki Declaration and the STROCSS2024 guideline: strengthening the reporting of cohort, cross-sectional, and case-control studies in surgery (Rashid et al. 2024).

### Patients

This study included women ≥ 18 years who required total or segmental mastectomies without immediate plastic reconstruction for non-metastatic primary breast cancer between January 1, 2015, and December 3, 2022, at MD Anderson Cancer Center. All consecutive patients who met inclusion criteria were considered for analysis. Those without RIOT information and those who may have received adjuvant therapies outside our institution were excluded. Also, those who did not receive rocuronium and those who underwent redo mastectomy for other oncological reasons or indications (i.e., emergency surgeries or biopsies) were excluded from the study. We also excluded patients who were pregnant, those admitted to the intensive care unit within 2 h after the procedure ended, those who underwent delayed reconstructive surgeries, and those who died within the first 30 postoperative days. In our institution, mastectomies are performed under an enhanced recovery after surgery program that emphasizes multimodal analgesia, minimization of opioid use, postoperative nausea and vomiting multimodal prophylaxis, early removal of urinary catheters, early feeding and mobilization, and early discharge. The primary medical oncological team made the decision on adjuvant therapies.

### Exposure

Patients were grouped according to the neuromuscular blocker reversal agent administered during surgery (i.e., into neostigmine or sugammadex groups). The decision of which neuromuscular blockade reversal drug to give was primarily based on the anesthesiologists’ clinical judgment.

### Endpoints

The study’s primary endpoint was time to RIOT, whether it was hormone therapy, chemotherapy, or radiotherapy—whichever was given first. Secondary endpoints included RIOT within 90 or 180 days, length of hospital stay, and 30-day hospital readmission.

### Variables and definitions

Demographic characteristics included age, body mass index, and race or ethnicity. The American Society of Anesthesiologists physical status assessed preoperative physical status. Data about neoadjuvant chemotherapy and radiotherapy 90 and 180 days before surgery and staging at the time of surgery were also collected (Chavez-MacGregor et al. 2016). The type of surgery was divided into segmental or total mastectomies. The years in which patients underwent surgery were classified into four periods: 2015–2016, 2017–2018, 2019–2020, and 2021–2022. This approach was taken to account for secular trend bias, temporal changes in sugammadex availability, and changes in oncological practices that may influence RIOT. The length of hospital stay and 30-day readmissions were recorded.

### Statistical analysis

Patients’ demographics and the status of RIOT were summarized using descriptive statistics. The Wilcoxon rank-sum test was used to compare location parameters of continuous distributions between patient groups. The *χ*^2^ test or Fisher exact test assessed associations between two categorical variables. The covariates that had a *P*-value of less than 0.10 from the univariable analysis and those known to clinically impact RIOT were included in the multivariate models. We evaluated for collinearity between independent variables to assess for violation in the regression analysis. A multivariable logistic regression model was fitted to estimate the effects of covariates on the status of any RIOT. An additional analysis of the time to RIOT was done through the Kaplan–Meier method; a univariable analysis was performed using the log-rank test for categorical variables, whereas a univariable Cox proportional hazards model was used for continuous variables. Prior evidence suggests a delay in chemotherapy initiation of longer than 90 days was clinically relevant (Chavez-MacGregor et al. 2016) Thus, at the significance level of 0.05, assuming a RIOT 90 days of 0.91 and considering an increase in the rate of 10% of RIOT clinically relevant, a minimum of 308 subjects were needed to achieve 80% power. *P* < 0.05 was considered statistically significant. Statistical software SAS 9.4 (SAS, Cary, NC, USA) was used for all the analyses.

## Results

**Fig. 1:**
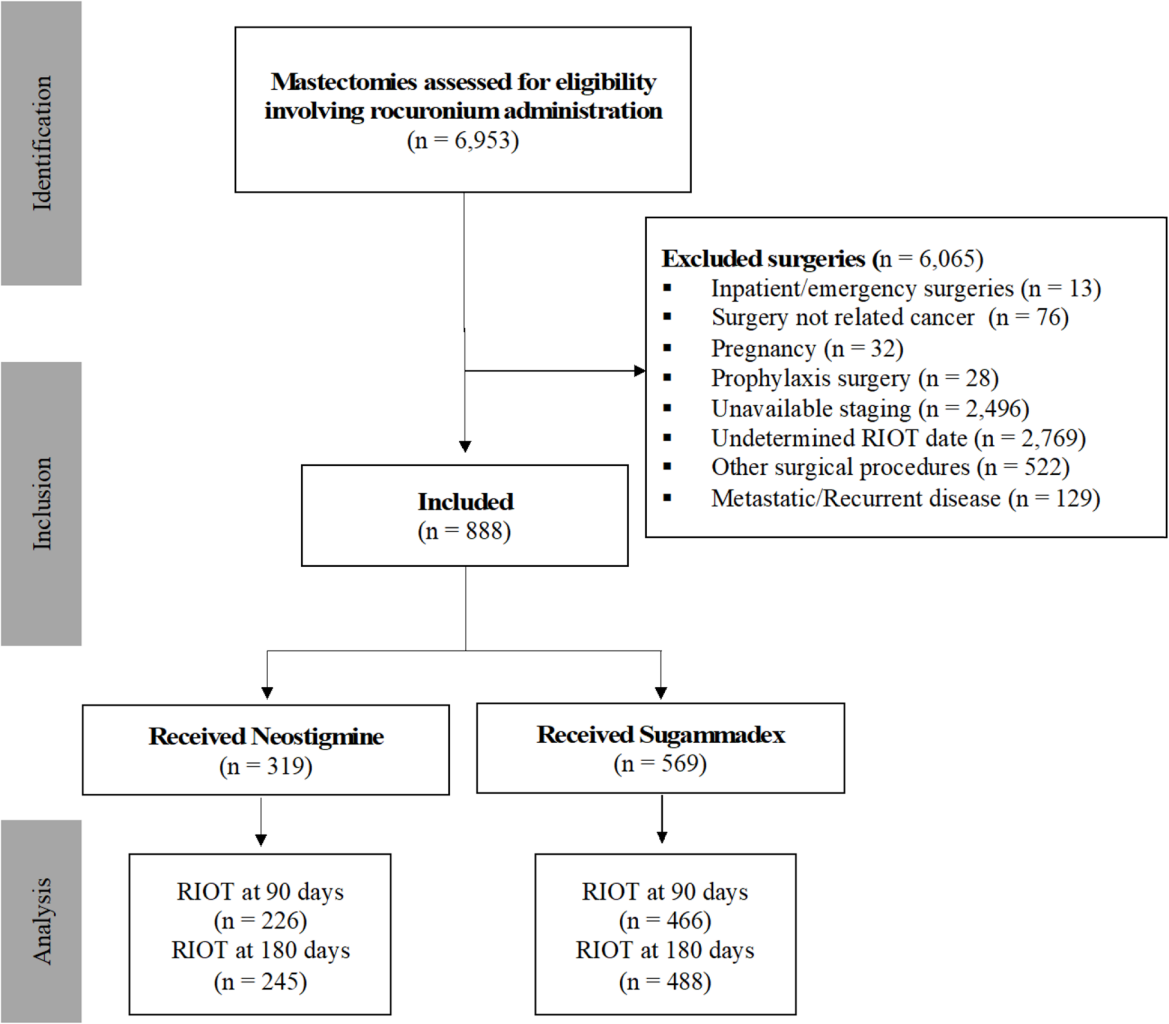
Flow diagram of the study

In this study, 6933 women who underwent breast surgery were screened for eligibility; 888 met the study criteria (Fig. 1). Of these, 319 patients received neostigmine, and 569 were treated with sugammadex.

The baseline clinical and intraoperative characteristics are summarized in Table 1. Women who received sugammadex were significantly younger than those treated with neostigmine (53 years, *IQR*: 45–85, vs. 56 years, *IQR*: 47–65; *P* = 0.013). While the difference was statistically significant, we do not consider it clinically relevant when considering cancer diagnosis and the choice of neuromuscular blockade reversal drug given. Other demographic variables did not differ significantly.

**Table 1:** Baseline clinical and intraoperative characteristics

	**Neostigmine (*n* = 319)**	**Sugammadex (*n* = 569)**	**Overall (*n* = 888)**	***p*-value**	
**Age, years**	56.0 (47.0–65.0)	53.0 (45.0–85.0)	54.0 (46.0–64.0)	0.013	
**BMI, kg/m**^**2**^	28.9 (24.5–33.4)	28.1 (24.0–33.0)	28.4 (24.2–33.1)	0.505	
**Race or ethnicity**				0.866	
Asian	22 (6.9%)	36 (6.3%)	58 (6.6%)		
Black/African American	42 (13.2%)	72 (12.7%)	114 (12.9%)		
Hispanic/Latino	51 (16.1%)	106 (18.7%)	157 (17.7%)		
White/Caucasian	194 (61.2%)	343 (60.4%)	537 (60.7%)		
Other	8 (2.5%)	11 (1.9%)	19 (2.1%)		
**ASA physical status classification**				0.078	
0–2	61 (19.3%)	83 (14.7%)	144 (16.4%)		
3–4	255 (80.7%)	481 (85.3%)	736 (83.6%)		
**90-day neoadjuvant therapy**	87 (27.3%)	237 (41.7%)	324 (36.5%)	< 0.001	
**90-day neoadjuvant chemotherapy**	87 (27.3%)	236 (41.5%)	323 (36.4%)	< 0.001	
**90-day neoadjuvant radiotherapy**	1 (0.3%)	4 (0.7%)	5 (0.6%)	0.660	
**Cancer stage**				0.382	
0	49 (15.4%)	54 (9.5%)	103 (11.6%)		
I	139 (43.6%)	275 (48.3%)	414 (46.6%)		
II	87 (27.3%)	145 (25.5%)	232 (26.1%)		
III	44 (13.8%)	95 (16.7%)	139 (15.7%)		
**Type of mastectomy**				0.759	
Segmental	153 (48%)	279 (49%)	432 (48.6%)		
Total	166 (52%)	290 (51%)	456 (51.4%)		
**Surgery period**				< 0.001	
2015–2016	272 (85.3%)	52 (9.1%)	324 (36.5%)		
2017–2018	20 (6.3%)	276 (48.5%)	296 (33.3%)		
2019–2020	14 (4.4%)	97 (17%)	111 (12.5%)		
2021–2022	13 (4.1%)	144 (25.3%)	157 (17.7%)		

Continuous variables are displayed as median (*IQR*). Categorical variables are displayed as *n* (%)

*Abbreviations:* *ASA* American Society of Anesthesiologists, *BMI* body mass index

The proportion of women receiving neoadjuvant therapy within 90 days from surgery was significantly higher in the sugammadex group than in the neostigmine group (41.7% vs. 27.3%; *P* < 0.001). No statistically significant differences in cancer staging and type of surgery were identified between treatment groups.

Of note, a statistically significantly larger proportion of neostigmine patients were operated on during the 2015–2016 period (85.3% vs. 9.1%), while 90.8% of the sugammadex patients had surgery after those years (*P* < 0.001). This reflected the broader availability of sugammadex in our practice and the degree of comfort anesthesia providers have with its use over time. Regarding dosages, the median [IQR] for neostigmine and sugammadex was 3 mg (Maaren et al. 2019; Kunkler et al. 2023; Slamon et al. 2024) and 200 mg [150–200].

### Time to RIOT

**Fig. 2:**
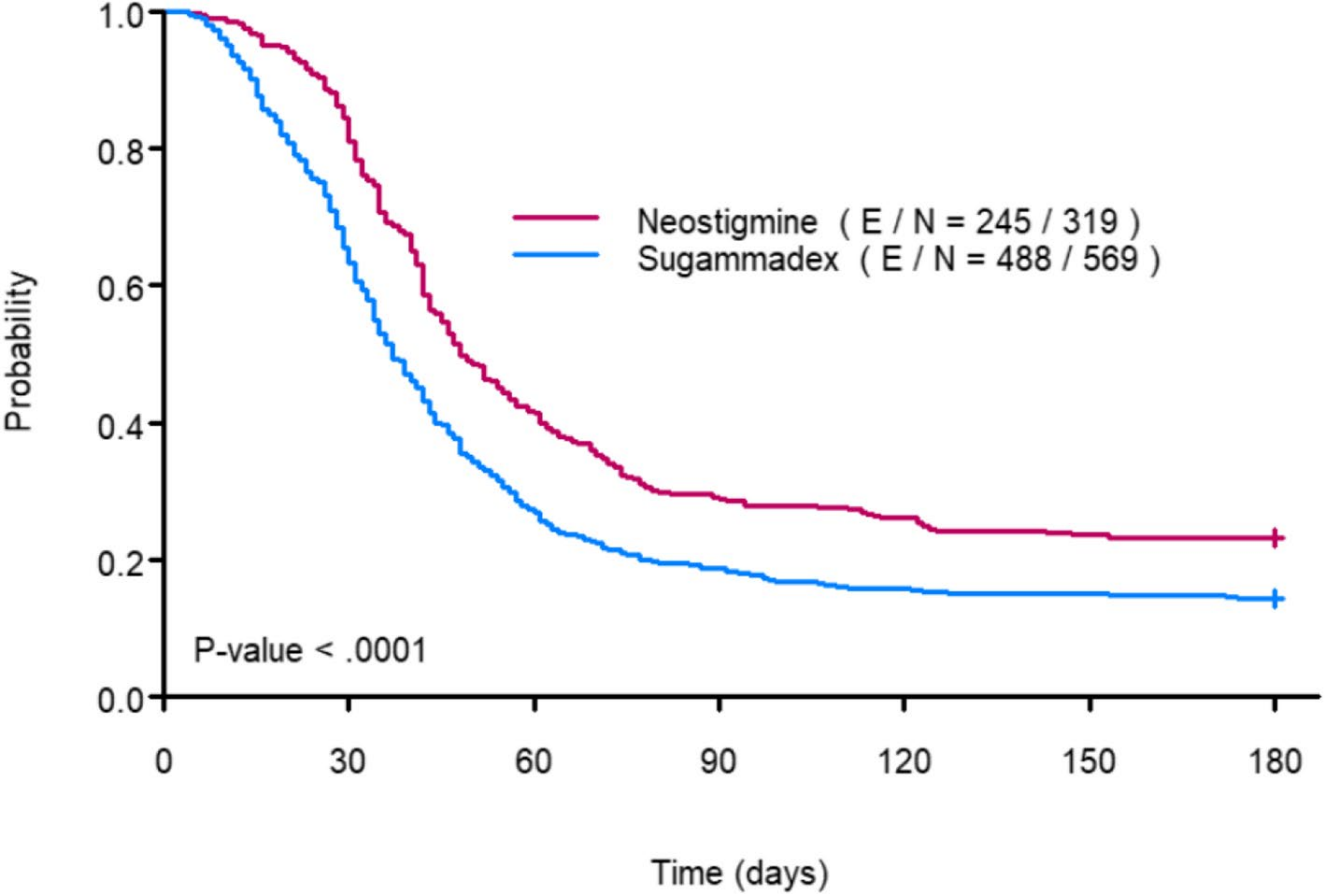
Time to RIOT. The figure depicts RIOT as time to event after mastectomy for breast cancer resection according to the neuromuscular blocker reversal agent administered during surgery. The time to RIOT was significantly lower when sugammadex was administered to revert the neuromuscular block. RIOT return to intended oncological therapy; E, event (RIOT); N, number of patients

The Kaplan–Meier curves in Fig. 2 show the impact of neostigmine and sugammadex on time to RIOT. The univariable analysis demonstrated that the median time to RIOT was shorter in the sugammadex group (median 37 days, 95% *CI*: 35–41) than in neostigmine patients (median 48 days, 95% *CI*: 45–56; *P* < 0.001). The multivariate Cox proportional hazards model showed that the administration of sugammadex did not statistically significantly influence time to RIOT after adjustments for tumor stage, time of procedure, and year of surgery (*HR*: 1.21, 95% *CI*: 0.974–1.504, *P* = 0.085, Table 2).

**Table 2:** Multivariable analysis for time-to-RIOT

**Variable**		**Hazard ratio**	**95% CI for HR**		***p*-value**
**Stage**	**1 vs. 0**	1.552	1.188	0.0013	2.028
	**2 vs. 0**	1.654	1.247	0.0005	2.195
	**3 vs. 0**	1.728	1.275	0.0004	2.341
**Primary procedure**	**Segmental vs. total mastectomy**	1.559	1.344	< 0.0001	1.807
**Surgery period**	**2017/2018 vs. 2015** **/2016**	0.954	0.750	0.7029	1.214
	**2019/2020 vs. 2015/2016**	1.633	1.248	0.0003	2.137
	**2021/2022 vs. 2015/2016**	2.134	1.659	< 0.0001	2.745
**NMBD**	**Sugammadex vs. neostigmine**	1.210	0.974	0.0845	1.504

*NMBD* neuromuscular blockade agent, *CI* confidence interval, *HR* hazard ratio

### Return to intended oncological therapy

As shown in Table 3, the univariable analysis demonstrated that women who received sugammadex achieved RIOT at 90 days at a statistically significantly higher frequency (81.9%) than those in the neostigmine group (70.8%, *P* < 0.001). Specifically, a significant difference in the rate of administration of adjuvant therapy—either chemotherapy or adjuvant—was observed, as 71.0% of the sugammadex patients received those treatments while only 39.2% of the neostigmine patients did (*P* < 0.001).

**Table 3:** 90 and 180 RIOT events

	**Neostigmine (*n* = 319)**	**Sugammadex (*n* = 569)**	**All (*n* = 888)**	***p*-value**
**90-day RIOT**				
**Overall treatment**	226 (70.8%)	466 (81.9%)	692 (77.9%)	< 0.001
** Chemotherapy**	125 (39.2%)	404 (71.0%)	529 (59.6%)	< 0.001
**Radiotherapy**	113 (35.4%)	236 (41.5%)	349 (39.3%)	0.076
**180-day RIOT**				
**Overall treatment**	245 (76.8%)	488 (85.8%)	733 (82.5%)	< 0.001
**Chemotherapy**	244 (76.5%)	452 (79.4%)	696 (78.4%)	0.306
**Radiotherapy**	118 (37.0%)	276 (48.5%)	394 (44.4%)	< 0.001

*RIOT* return to intended oncological therapy

When the upper limit for considering RIOT was extended to 180 days, the univariate analysis also showed that the proportion of patients that achieved this outcome remained statistically significantly higher in the sugammadex group (85.8%) than in the neostigmine group (76.8%; *P* < 0.001). Interestingly, within 180 days, the difference in RIOT was primarily due to a higher use of adjuvant radiotherapy in the sugammadex group (48.5% vs. 37.0%, *P* < 0.001).

The multivariable logistic regression model demonstrated that after adjustments for age at surgery, cancer stage, and type of surgery, the administration of sugammadex was not associated with higher odds of RIOT at 90 days over neostigmine administration (*OR*: 1.175, 95% *CI*: 0.685–2.0140, *P* = 0.559). Similarly, compared with neostigmine, RIOT at 180 days was not significantly affected by the administration of sugammadex (*OR*: 0.846, 95% *CI*: 0.471–1.519, *P* = 0.575, Table 4).

**Table 4:** Multivariate analysis on 90-day and 180-day return to intended oncological therapy

	**90-day RIOT**					**180-day RIOT**				
	**OR estimate**		**95% CI for OR**		***p*-value**	**OR estimate**		**95% CI for OR**		***p*-value**
**Age, in years**	1.005		0.99	1.019	0.5412	-		-	-	-
**Cancer staging**										
I vs 0	3.213		1.909	5.41	< 0.0001	3.939		2.281	6.802	< 0.0001
II vs 0	2.867		1.643	5.004	0.0002	4.179		2.303	7.583	< 0.0001
III vs 0	4.354		2.27	8.351	< 0.0001	6.731		3.271	13.85	< 0.0001
**Mastectomy (segmental vs total)**	3.415		2.341	4.983	< 0.0001	3.938		2.597	5.971	< 0.0001
**Surgery period**										
2017/2018 vs 2015/2016	1.012		0.582	1.76	0.9671	1.443		0.795	2.618	0.2282
2019/2020 vs 2015/2016	3.694		1.636	8.343	0.0017	4.68		1.901	11.518	0.0008
2021/2022 vs 2015/2016	7.759		3.285	18.326	< 0.0001	16.146		5.237	49.779	< 0.0001
**Sugammadex vs neostigmine**	1.175		0.685	2.014	0.5587	0.846		0.471	1.519	0.5752

### Length of stay and 30-day hospital readmission

The univariable analysis showed that the median length of stay was not statistically significantly different between the two groups (sugammadex: 1.0 day, *IQR*: 0.00–1.00 vs. neostigmine: 1.0 day, *IQR*: 0.00–1.00; *P* = 0.168). The analysis also showed no difference in the incidence of 30-day hospital readmission between sugammadex-treated (*n* = 3, 0.5%) and neostigmine-treated patients (*n* = 1, 0.3%; *P* = 1.000).

## Discussion

In breast cancer patients, omitting or delaying adjuvant therapies negatively affects several oncological outcomes, including disease-free survival and overall survival rates (Chavez-MacGregor et al. 2016; Cold et al. 2005; Ke-Da et al. 2013; Lohrisch et al. 2006). Our study evaluated the impact of two routinely used neuromuscular relaxant reversal agents on RIOT-related outcomes and hence may not influence oncological outcomes. Patients who received sugammadex had a higher RIOT rate within 90 and 180 postoperative days than those who received neostigmine. Time to RIOT was also faster for sugammadex patients by 11 days compared with neostigmine patients. However, after adjusting for confounders, we observed that the use of sugammadex did not independently influence RIOT incidence nor the time to RIOT in women undergoing breast cancer surgery. It is important to consider that the shorter time to RIOT in the sugammadex group may not be directly attributable to the drug itself but rather to changes in oncology practice following the publication of two influential articles in 2016 (Chavez-MacGregor et al. 2016; Raphael et al. 2016).

Our work was motivated by prior evidence indicating that a single perioperative anesthetic intervention, such as the intraoperative infusion of ropivacaine to alleviate postoperative pain, reduced the time to chemotherapy in women undergoing gynecological surgeries (Hayden et al. 2020). However, time to RIOT and its incidence did not differ in patients with colorectal cancer randomly assigned to receive thoracic epidural or intravenous opioid analgesia for postoperative pain control (Falk et al. 2021). Similarly, a sub-analysis of a randomized controlled trial among women with breast cancer randomly allocated to total intravenous anesthesia or inhalatory volatile anesthesia showed no differences in time to RIOT and time to completion of intended oncological therapy (Ní Eochagáin et al. 2018). More relevant to our work, a retrospective study in 1643 adult patients with gastric cancer demonstrated that high dosages of intermediate-acting neuromuscular blocker agents independently predicted the time to RIOT, which could be explained by an increase in complications associated with residual neuromuscular blockade (Niu et al. 2020).

Studies have shown that sugammadex use positively influences perioperative outcomes such as time to extubation, ileus, length of stay, and postoperative pulmonary complications without a major increase in side effects (Ruetzler et al. 2022; Suleiman et al. 2023; Fortier et al. 2015; Vaghiri et al. 2023). For example, in one large multicentric retrospective study that included 83,250 patients, sugammadex administration was associated with a 14% increase in the odds of discharge after surgery but without differences in intensive care unit admission and 30-day readmission (Suleiman et al. 2023). In that study, postoperative respiratory complications were reduced by 16.0% (Suleiman et al. 2023).

In women requiring mastectomies, delay or inability to RIOT is impacted by several factors, most notably postoperative complications (Huttunen et al. 2022; Eck et al. 2015) For instance, infections and reoperations negatively impacted the time to chemotherapy in a Finnish cohort of women who underwent mastectomies with or without immediate reconstruction (Huttunen et al. 2022). Therefore, the lack of independent association between the use of sugammadex and RIOT-related outcomes in our study could be explained by our cohort of patients’ relatively low postoperative morbidity as evidenced by the short length of stay and low incidence of 30-day readmission.

Our study has several limitations. First, due to its retrospective nature, several confounders were not accounted for, such as frailty or more comprehensive indices of morbidity (i.e., Elixhauser or Charlson comorbidity index) and residual neuromuscular blockade not available in our dataset or deciding when to start adjuvant therapy based on access to care. Such variables should be incorporated into future observational studies. In addition, patients without RIOT information and those who may have received adjuvant therapies outside our institution were excluded. While women in the RIOT group were slightly but statistically significantly younger, we do not believe that 3 years of age difference can explain differences in RIOT results. The combination of early cancer detection and an increased use of sugammadex in recent years could explain our finding (Zhang-Petersen et al. 2024) Second, we consider neostigmine as an active comparator. However, as sugammadex was introduced in 2015 and was rapidly implemented as a standard of care practice at our institution after 2016, the number of patients receiving neostigmine was limited and negatively impacted the power of the statistical analysis. Third, we did not address the postoperative complications individually, including residual neuromuscular blockade. It is possible to speculate that patients who developed severe postoperative complications may have never received adjuvant therapies and hence were excluded from our study. In other terms, a low baseline event rate, and particularly a low rate of postoperative pulmonary complications, which are the key hypothesized mediator of causality, would make women with breast cancer a less-than-ideal population to study since the signal-to-noise ratio will be low, as evidenced by the low readmission rate and short length of stay. Along these lines, our work included a low-risk surgical population, where the choice of reversal may not significantly affect postoperative morbidity. Further, only a minority of patients in this group are likely to receive muscle relaxation and reversal agents, limiting the generalisability of findings. Fourth, our study did not include data from patients who underwent extensive breast reconstructions. This was motivated by the higher burden of postoperative symptoms after breast reconstruction, such as general activity and fatigue, and a shift in the number of those procedures in recent years which may have influenced the decision of early RIOT (Wang et al. 2024) Last, we did not evaluate the economics of administering sugammadex on a low-risk surgical population of patients. We can speculate that in this surgical population, the administration of sugammadex may not be justified from a cost-effectiveness perspective.

In conclusion, the administration of sugammadex, compared with neostigmine administration, was not associated with improvements in RIOT incidence and time to RIOT in this retrospective study assessing patients undergoing surgery for breast cancer. Future work investigating the impact of neuromuscular reversal choice on higher-risk surgery is needed. Prospective studies will be needed to confirm our findings in breast cancer patients and those with other malignancies.

## Data Availability

No datasets were generated or analysed during the current study.
